# Staying Streetwise: Accurate Judgments of Approaching Aggression in Older Age

**DOI:** 10.5964/ejop.v14i1.1369

**Published:** 2018-03-12

**Authors:** Liam Paul Satchell, Lucy Akehurst, Paul Hayden Morris, Claire Nee

**Affiliations:** a Department of Psychology, University of Portsmouth, Portsmouth, United Kingdom; Department of Psychology, Webster University Geneva, Geneva, Switzerland; University of Liverpool, Liverpool, United Kingdom

**Keywords:** aggression detection accuracy, intimidation perception, older adults

## Abstract

The extant literature has generally demonstrated that young adults can detect the trait aggression of another person with limited information. However, there is little research that investigates the life course persistence of aggression detection accuracy. Here, we aimed to explore the accuracy of older adults at detecting potential aggressors. Thirty-nine older adults (M = 71.49, SD = 7.59) and eighty-seven young adults (M = 20.24, SD = 1.74) made intimidation judgments, via video recordings, for nine people (targets). ‘Aggression detection accuracy’ was shown in the relationship between the intimidation judgments made by participants and the targets’ responses to the Buss-Perry Aggression Questionnaire. Both age groups were highly accurate in their recognition of trait aggression and accuracy was maintained into older age, with no difference in accuracy between the older and young adults. There was, however, more variability in the ratings given by the older adults compared to the young adults, suggesting less consensus in judgment for the older compared to the young group. Overall, the participants in this study were highly accurate at detecting trait aggression. There was no difference in average aggression detection between older and young adults but there was in sample agreement. These results are discussed in the context of age effects on intimidation, as well as research in accurate aggression detection.

For some time the consensus in the literature was that older adults were more afraid of becoming a victim of crime than young adults (Clarke & Lewis, 1982; Clemente & Kleiman, 1977; Kennedy & Silverman, 1985; Moeller, 1989; Ortega & Myles, 1987). However, more recently research has demonstrated that the relationship between age and fear of crime is not so clear, and the relationship is mediated by many factors; including the crime type, gender of the respondents and participants’ belief in being able to defend themselves (see Acierno, Rheingold, Resnick, & Kilpatrick, 2004; Beaulieu, Dubé, Bergeron, & Cousineau, 2007; Jackson, 2009; Kappes, Greve, & Hellmers, 2013; Oh & Kim, 2009). Despite the evidence that fear or intimidation is situational, there is little experimental research investigating older adults’ detection of potentially dangerous others. It could be that fear of crime is relative to an individual’s ability to detect potentially dangerous others with ease. Previous research has demonstrated that simply watching someone walk communicates aspects of dispositional aggression. In particular young adults were more accurate than teenagers at detecting aggression through intimidation ratings (Satchell, Akehurst, & Morris, 2017). The accuracy of older adults, when presented with a similar task, has not yet been investigated.

It is important to make quick, but also accurate, judgments of the danger posed by others. Even with limited interaction, people are generally good at detecting the trait properties of others (Cheek, 1982; Funder, 1980; Letzring, 2015; McCrae, 1982; Vazire & Mehl, 2008) and previous work suggests that accuracy of ‘social perceptions’ is robust into older age (for an overview see; Freund & Isaacowitz, 2014). Funder’s (1999) Realistic Accuracy Model (RAM) focusses upon the *availability* of *relevant* information regarding targets’ traits, which can then be *detected* by a judge to *utilize* for an accurate personality judgment. This focus on the detection of traits being as much a property of the targets as it is the judges is important, and often overlooked. This is especially important in the context of approaching people, as the information elicited by targets changes in quantity and quality as they approach a judge. As an unknown person approaches, it will frequently be their walk style and their body outline that are *available* (to use the RAM term) to a judge first. Most of the work on the judging of malevolent attributes of others relies on photographs of faces (such as; Geniole, Molnar, Carré, & McCormick, 2014; Hehman, Flake, & Freeman, 2015; Hehman, Leitner, & Gaertner, 2013) and does not include other information potentially relevant to trait aggression, which would be available from an approaching person (such as walk style or body appearance). For example, recent research has shown that there is information, relevant to aggression, in how someone walks (Satchell, Morris, Mills, et al., 2017). If older adults can use gait information to detect trait aggression, much like young adults can (Satchell, Akehurst, & Morris, 2017; Satchell, Morris, Akehurst, & Morrison, 2017), then they will be able to make ‘approach or avoid’ responses when a stranger is walking towards them.

Research has shown that adults are able to accurately judge the dispositional aggression of a target person from simply viewing a photograph of their face (Carré, McCormick, & Mondloch, 2009). Boshyan, Zebrowitz, Franklin, McCormick, and Carré (2013) have even demonstrated that older adults are as accurate as young adults in detecting targets’ performance in a Point Subtraction Aggression Paradigm (Carré & McCormick, 2008) from viewing brief presentations (3 seconds) of photographs of faces. If older adults are accurate at detecting the traits of others from viewing photographs of faces, then it is possible they will also be as accurate, if not more accurate, when judging realistic presentations of a target; a walking person.

For the current experiment, we expected the judgments of intimidation would relate to each target’s trait aggression and refer to this relationship as *accuracy* (see Funder, 2012). Given that older adults have been shown to be as accurate as young adults in detecting aggression from photographs of faces, we were also expecting this to be the case in the current experiment. In fact, as our participants were presented with more realistic information than simply the face of a target, we expected our older and young adults’ judgments of aggression would be more accurate than those of previous research that only presented still photographs of faces.

## Method

### Participants

The thirty-nine older adults (27 females and 12 males, *M_Age_* = 71.49, *SD_Age_* = 7.59, Min_Age_ = 59, Max_Age_ = 91) were an opportunity sample recruited during public lectures delivered to members of an Aging Network research group. These were individuals who engage with academic events hosted by a University. Eighty-seven undergraduate young adults (73 females and 14 males, *M_Age_* = 20.24, *SD_Age_* = 1.74, Min_Age_ = 18, Max_Age_ = 28) were recruited as a comparison group. The young adults were recruited and took part in a similar forum to the older adult sample; in an undergraduate lecture.

### Materials

We filmed the targets walking at their preferred speed for 10 seconds on a treadmill. For the purpose of keeping the experiment efficient and retaining the engagement of all participants, a sub-set of nine targets (from our database of 23) were chosen for use in this study. These nine targets (5 females and 4 males, *M*_Age_ = 20.67, *SD*_Age_ = 2.40, Min_Age_ = 18, Max_Age_ = 24) were randomly selected using a random number generator.

We used the Physical Aggression subscale from the Buss-Perry Aggression Questionnaire (Buss & Perry, 1992) to capture our targets’ trait aggression. This subscale, when analysed using advised revisions (Bryant & Smith, 2001), gives a score between 3 and 21 and our nine targets were reasonably spread in the range of possible scores (*M*_Aggression_ = 7.11, *SD*_Aggression_ = 5.09, Min_Aggression_ = 3, Max_Aggression_ = 15). This subscale was chosen, as it is most pertinent to interpersonal aggression. The Buss-Perry Questionnaire is well established in the aggression literature and has been used with various forensic and non-forensic populations to predict historic and laboratory expressions of aggression (see; Archer & Webb, 2006; Bryant & Smith, 2001; Diamond, 2006; Diamond, Wang, & Buffington-Vollum, 2005; García-León et al., 2002; O’Connor, Archer, & Wu, 2001). The physical aggression subscale has internal reliability of between α = .84 and α = .86 across three samples used by Bryant and Smith (2001). Using 5 samples, Webster et al. (2014) showed that the Buss-Perry Aggression Questionnaire – Short Form is internally reliable, shows strong test-retest reliability and predicts behavioural measures of aggression.

### Procedure

Participants took part in groups but made their ratings privately on written response sheets, with instructions not to communicate about the ratings to any other participants. The targets were presented on a screen (measuring approximately 3m × 2.5m) and for each presentation (k = 3) the order of presentation of the nine targets was randomised. After the presentation of each target, the participants were given as much time as they required to make ratings, on 9-point Likert scales, of how *intimidating-not intimidating*, *friendly-unfriendly* and *masculine-feminine* they perceived each target to be.

### Analyses

We conducted our statistical analyses in two different ways. It is typical of interpersonal perception experiments of this type to calculate the average rating received by each target and then correlate this value with the targets’ traits to demonstrate whole group accuracy (see *Ratings received by targets* section of the Results). We included this analysis for the purpose of comparison with other research but this ‘nomothetic’ approach to analysing judgment data has faced criticism. Such analysis ignores the individual variation in judge ability and falsely increases the size of correlations (see; Brand & Bradley, 2012; Hirschmüller, Egloff, Schmukle, Nestler, & Back, 2015; Kolar, Funder, & Colvin, 1996; Monin & Oppenheimer, 2005).

As such, we also adopted the recommended ‘idiographic’ analysis (Kolar et al., 1996; that is referred to as the ‘average of correlations’ by Monin & Oppenheimer, 2005), where an accuracy value is calculated for each participant. This is achieved by computing the correlation between judgments of each individual judge and the targets’ properties, thus producing a value between 1 (accurate; a target rated as more intimidating is more aggressive) and -1 (inaccurate; a target rated as more intimidating is less aggressive) and the size of each value denotes the strength of agreement (0 being random performance, ±1 being perfect [in]accuracy). The idiographic analysis allows us to report the distribution of judge variability, as well as to test for the effects of age on accuracy (see *Accuracy of participants* section of the Results).

## Results

### Ratings Received by Targets

The average intimidation ratings the targets received positively correlated with the targets’ trait aggression, with notably large effects (young adults, *r*(9) = .85, 95% CI [.37, .99], *p* = .004; older adults, *r*(9) = .82, 95% CI [.48, .96], *p* = .007). Thus, both the older and young adults demonstrated very strong accuracy at detecting trait aggression through intimidation ratings (there was no meaningful difference between the two groups’ accuracy correlations; *z* = .17, *p* = .865.) Further, we found that the average rating received by the targets from the older and young adults strongly correlated (*r*(9) = .79, 95% CI [.46, 1.00], *p* = .012) suggesting similar allocation of intimidation ratings.

In all measures in psychology it is typical to report both the mean and the standard deviation of a distribution. It is somewhat surprising that similar research does not report the variation in ratings received by the targets. Variance in judgments demonstrates more guess work or a lack of consensus about the matter being judged. By calculating the standard deviation (σ) of intimidation ratings received by each target, we were able to test for differences in spread of intimidation judgments between the age groups. We found that the variation in intimidation ratings differed between age groups (*t*(8) = 2.70, *p* = .027, *d* = .76) with the older adults being more varied in their ratings (*M*_σ_ = 2.02, *SD*_σ_ = .28) than the young adults (*M*_σ_ = 1.78, *SD*_σ_ = .35). The implication of these results is that whilst, on average, the groups performed similarly, there was less consistency in ratings for the older adults compared to the young adults.

### Accuracy of Participants

**Figure 1:**
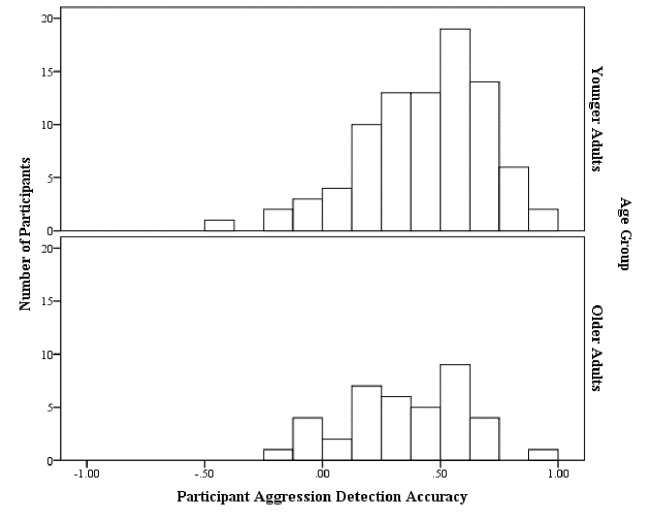
The distribution of participant accuracy (individual correlations) for each age group.

Given the variation in ratings demonstrated above, it was important to consider the distribution of judge accuracy (see Figure 1). Thus, we report the distribution of individual accuracy correlations (*r,* where *r* = 1 is perfect accuracy, *r* = -1 perfect inaccuracy and *r* = 0 random responding). There was no real difference in accuracy^i^ between the older adults (*M_r_* = .35, *SD_r_* = .26, Min*_r_* = -.24, Max*_r_* = .91, Skewness = -.24 [*SE* = .38], Kurtosis = -.35 [*SE* = .74]) and young adults (*M_r_* = .43, *SD_r_* = .27, Min*_r_* = -.48, Max*_r_* = .92, Skewness = -.77 [*SE* = .26], Kurtosis = .84 [SE = .51]) (*t*(124^ii^) = 1.61, *p* = .110, *d* = .31). It is important to note that accuracy was generally good across both age groups.

It is notable that some participants were extremely accurate (six participants achieved *r*>.80, five of whom were in the young adults group). In some cases, there were very small differences in the self-report measures of the targets (in some cases a one point difference in their self-reported aggression), yet some participants were able to detect these subtle differences.

## Discussion

In this study, we found that older adults were as accurate as young adults at detecting the trait aggression of our targets. We found that the ratings received by the targets from both the older and young adult groups correlated strongly with each other and with the trait aggression of the targets. In fact, our measures showed that both age groups were reasonably good at indexing trait aggression through intimidation ratings, with some participants being notably good at using their intimidation ratings to reflect even minor differences in trait aggression for the targets. There was some evidence of a negative correlation between age and accuracy, but the sample lacked participants between the ages of 28 and 59 years so this finding should be treated with caution. Our categorical analyses (young adults vs. older adults) demonstrated no difference between the groups in terms of accuracy. The only clear difference between the groups was in the spread of ratings received by the targets. The young participants showed more consensus in intimidation ratings than the older adults. Whilst this did not impact upon accuracy (it is apparent that the discrimination between more and less dangerous targets is constant between groups), it may show that there is less specificity in what intimidation means to the older adults.

The fact that intimidation ratings relate to the ordering of the targets’ aggression is interesting and somewhat surprising. Given that some of the self-report aggression ratings made by the targets were very similar, it is unexpected that some participants achieved such high accuracy. It is also important that, whilst the relationship is implicit, intimidation is not a direct reference to aggression. Boshyan et al. (2013) asked participants to form explicit aggression judgments about photographs of targets and they told their participants about the paradigm through which the aggression values for each target had been attained (a computer task called the Point Subtraction Aggression Paradigm). In the current study, we did not inform participants of our intentions to test them for accuracy thus allowing them to make a general judgment of intimidation. Our participants did not know that their judgments would be compared with the self-reported aggression of the targets. Interestingly, the current findings replicated the findings of Boshyan et al. (2013) and found evidence of strong accuracy in the older population^iii^.

The inconsistencies in the relationship between age and fear of crime could be due to individual differences. As previous research shows, the idiosyncrasies of respondents, context and crime type all have an effect on fear of crime judgments in older adults (Acierno et al., 2004; Beaulieu et al., 2007; Jackson, 2009; Kappes et al., 2013; Oh & Kim, 2009). It is possible that older adults who are reasonably accurate at detecting potential dangers may well be those adults who are less fearful of crime. Asymmetry between feelings of intimidation and genuine danger could lead to psychological and physical health consequences. A growing body of literature has demonstrated that how much an individual fears crime in their local area affects the time they spend walking (Foster, Knuiman, Hooper, Christian, & Giles-Corti, 2014), cycling (Kramer, Maas, Wingen, & Kunst, 2013) and how much time older adults spend engaging in healthy activity programs (Dawson, Hillsdon, Boller, & Foster, 2007). Perhaps dissemination of research findings that find that older people’s judgments of dangerous others are largely accurate may provide individuals with encouragement to engage with their environment. Equally, it could be the case that those who feel more intimidated in general are those with poor accuracy. Future research might usefully evaluate the possibility of improving accuracy through training.

### Limitations and Future Directions

We were interested in demonstrating that older adults can be accurate at judging danger via realistic presentations of targets. As our stimuli (videos) were time-consuming to present, only nine randomly-selected targets (from a larger sample) were presented to participants. Future work could consider more targets and perhaps, rather than presenting targets one at a time, they could be presented in groups (something that many of the older participants self-reported as being important for their everyday judgments of intimidation).

Future work could also seek to study larger and more varied populations of both the older and younger participants. Our current sample is based on specific populations (students and older adults who are comfortable enough to travel to a public talk), moving beyond these populations may find different results. In expanding the population there could be more attention paid to the aforementioned idiosyncratic fear of crime aspects affecting older adults (gender of the respondents and participants’ belief in being able to defend themselves; Acierno et al., 2004; Beaulieu et al., 2007; Jackson, 2009; Kappes et al., 2013; Oh & Kim, 2009). The study could also be conducted more privately, testing one participants one at a time. Methodologically similar research on student populations finds complementary findings to the current study (Satchell, Morris, Akehurst, & Morrison, 2017), it is not clear what effect, if any, conducting the study in a group may have had on the participants.

It would also be of interest to investigate the detection of non-physical risk. As Acierno et al. (2004) note, perceived risk of property crime is more relevant for older adults than a physical crime, so perhaps a test of accuracy in detecting a target person’s propensity to commit acquisitive crime could be of interest (for example, in the context of doorstep crime).

### Conclusion

In sum, older adults were as accurate as young adults at detecting the trait aggression of walking targets using ratings of intimidation. Previous research has suggested that experience is essential for the acquisition of accurate intimidation judgments (Satchell, Akehurst, & Morris, 2017) and so it is reasonable to suggest that accuracy does not decline as people attain experience throughout their lives. Whilst the current findings need to be replicated, with more participants, more targets and a greater age range of participants and targets, they convey a positive message for older adults in that their gut reactions pertaining to the threat posed by approaching strangers are likely to be accurate.
